# Reproduction in deep‐sea vent shrimps is influenced by diet, with rhythms apparently unlinked to surface production

**DOI:** 10.1002/ece3.9076

**Published:** 2022-07-17

**Authors:** Pierre Methou, Chong Chen, Hiromi Kayama Watanabe, Marie‐Anne Cambon, Florence Pradillon

**Affiliations:** ^1^ Univ Brest, CNRS, Ifremer, UMR6197 Biologie et Ecologie des Ecosystèmes marins Profonds Plouzané France; ^2^ X‐STAR, Japan Agency for Marine‐Earth Science and Technology (JAMSTEC) Yokosuka Japan

**Keywords:** biological rhythms, crustacean, deep sea, hydrothermal vent, reproduction, seasonality, trophic ecology

## Abstract

Variations in offspring production according to feeding strategies or food supply have been recognized in many animals from various ecosystems. Despite an unusual trophic structure based on non‐photosynthetic primary production, these relationships remain largely under‐studied in chemosynthetic ecosystems. Here, we use *Rimicaris* shrimps as a study case to explore relationships between reproduction, diets, and food supply in these environments. For that, we compared reproductive outputs of three congeneric shrimps differing by their diets. They inhabit vents located under oligotrophic waters of tropical gyres with opposed latitudes, allowing us to also examine the prevalence of phylogenetic vs environmental drivers in their reproductive rhythms. For this, we used both our original data and a compilation of published observations on the presence of ovigerous females covering various seasons over the past 35 years. We report distinct egg production trends between *Rimicaris* species relying solely on chemosymbiosis—*R. exoculata* and *R. kairei*—and one relying on mixotrophy, *R. chacei*. Besides, our data suggest a reproductive periodicity that does not correspond to seasonal variations in surface production, with substantial proportions of brooding females during the same months of the year, despite those months corresponding to either boreal winter or austral summer depending on the hemisphere. These observations contrast with the long‐standing paradigm in deep‐sea species for which periodic reproductive patterns have always been attributed to seasonal variations of photosynthetic production sinking from the surface. Our results suggest the presence of an intrinsic basis for biological rhythms in the deep sea, and bring to light the importance of having year‐round observations in order to understand the life history of vent animals.

## Graphical abstract

Diagrams summarizing occurrence of reproductive stages of the studied Rimicaris shrimp species over different samplings realized between 1985 and 2018 according to the year period

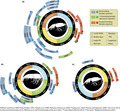

## 1 INTRODUCTION

Relationships between feeding strategies and reproduction have been extensively studied for a number of animal phyla in various ecosystems (Almeida et al., 2018; Broderick et al., 2003; Gutiérrez et al., 2020; Lin & Shi, 2002; Meiri et al., 2012; Pierotti & Annett, 1990; Qian & Chia, 1991; Sibly et al., 2012; Tziouveli et al., 2011). However, all these studies are based on ecosystems where photosynthetic primary production is the sole basis of food webs. A number of ecosystems on Earth, such as deep‐sea hydrothermal vents, cold seeps, and organic falls, rely on another primary organic source: chemosynthesis (German et al., 2011). These chemosynthesis‐based ecosystems are supported by microbial communities using chemical energy from geofluids to produce their organic matter. There, animals can derive nutrition from either chemosynthesis through intricate symbiotic associations or direct consumption of free‐living chemosynthetic microbes, sinking phytodetrital production from the surface, or a mixture of both, leading to the emergence of trophic networks often differing strikingly from those found elsewhere (Dubilier et al., 2008; Govenar, 2012; Sogin & Leisch, 2020). In this context, these environments are natural laboratories to study the relationships between reproduction and diet.

Although most studied species from deep‐sea chemosynthesis‐based ecosystems exhibit aperiodic reproductive patterns—referred to continuous in some studies—as seen in some alvinellid and polynoid polychaetes and several gastropod families (Faure et al., 2007; Jollivet et al., 2000; Marticorena et al., 2020; Tyler et al., 2008), seasonal cycles following variations in surface primary production have also been observed in many taxa including bathymodioline mussels and alvinocaridid shrimps (Copley & Young, 2006; Dixon et al., 2006; Tyler et al., 2007). Additionally, both aperiodic and seasonal reproductive patterns may be observed in different species belonging to the same family, like in the bythograeid crabs (Hilário et al., 2009; Perovich et al., 2003). Although the trophic strategy of these species is not always known in detail, seasonal reproduction has so far only been observed in those species capable of assimilating photosynthetic sinking particles, even if it constitutes a negligible part of their diet (Riou et al., 2010). Moreover, a clear link between surface productivity and reproductive patterns could be established in bythograeid vent crabs, with species inhabiting under highly oligotrophic waters (South Pacific Gyre) exhibiting aperiodic gametogenesis, while others living in regions with more seasonally fluctuating productivity showed corresponding reproductive patterns (Hilário et al., 2009; Perovich et al., 2003). Such examples have led to the hypothesis that the rhythm and periodicity of sinking photosynthetic matter are key time‐synchronizing cues for deep‐water animals exhibiting seasonal reproduction, signaling individuals to spawn at the same period of the year.

The symbiotic and congeneric shrimps *Rimicaris exoculata* and *R. kairei* are among the most iconic hot‐vent animals, with unique adaptations such as hosting symbiotic bacteria in their enlarged cephalothorax cavity and the fused “dorsal eye,” a novel sensory system representing a pinnacle of adaptation to vents (Zbinden & Cambon‐Bonavita, 2020). The entire family they belong to, Alvinocarididae, is endemic to vents and seeps with different taxa exhibiting various trophic regimes and various levels of reliance on symbiosis (Apremont et al., 2018; Gebruk et al., 2000; Rieley et al., 1999), constituting an ideal case for elucidating relationships between reproduction and diet in chemosynthesis‐based ecosystems. Species with different trophic strategies often co‐occur in the same vent fields, for example, *R. exoculata*, which relies on symbiosis and the mixotrophic *R. chacei*, allowing for a straightforward comparison between congeneric species. The symbiotrophic species such as *R. exoculata* and *R. kairei* exhibit greatly inflated cephalothoraxes and live in large aggregations around the hot fluid flows (Gebruk et al., 2000; Methou et al., 2020; Rieley et al., 1999; Streit et al., 2015; Van Dover, 2002), whereas the mixotrophic species *R. chacei* (Apremont et al., 2018; Gebruk et al., 2000; Methou et al., 2020) lacks the inflated cephalothorax and lives in smaller groups, hidden under mussels beds or interstices between sulfides and at a greater distance from the hot fluid sources (Methou et al., 2022).

Attempts to understand the reproduction of Rimicaris shrimps have so far been inconclusive due to contradicting evidences. As with most carideans (Correa & Thiel, 2003), *Rimicaris* shrimps are gonochoric, with the females brooding their eggs under the abdomen until the larva hatches (Hernández‐Ávila et al., 2021; Ramirez‐Llodra et al., 2000). While aperiodic reproduction was initially suggested from the examination of their reproductive tissues (Copley et al., 2007; Ramirez‐Llodra et al., 2000), very few ovigerous individuals were collected in over 35 years of seagoing expeditions, despite a relatively focused and repeated sampling effort (Copley et al., 2007; Komai & Segonzac, 2008; Ramirez‐Llodra et al., 2000; Shank et al., 1998; Vereshchaka, 1997; Williams and Rona, 1986). Brooding females were first found in small numbers in 2007 (Guri et al., 2012), and much greater numbers were recovered in 2014 (Hernández‐Ávila et al., 2021; Methou et al., 2019), around a restricted time period between January and March. These females were living within dense aggregations close to vent orifices (Hernández‐Ávila et al., 2021), refuting the previous hypothesis that brooding females would migrate to the vent periphery to protect their eggs from vent fluid, as observed for bythograeid crabs (Perovich et al., 2003) and *Kiwa* squat lobsters (Marsh et al., 2015). Taken together, these recent findings are suggestive of a seasonal reproductive cycle possibly linked to surface primary production, as shown in vent crabs, bathymodiolin mussels, and some other alvinocaridid shrimps (Copley & Young, 2006; Dixon et al., 2006; Tyler et al., 2007). However, the low primary productivity of oligotrophic surface waters and the resulting overall low export to the deep sea where these shrimps have been collected (−3500 m depth) (Harms et al., 2021; Pabortsava et al., 2017) challenge the plausibility of such a link.

Here, we compare reproductive outputs—that is, fecundities and egg volumes—of three congeneric vent shrimps with contrasting diets, using *R. exoculata* and *R. kairei* that are fully dependent on chemosymbiosis, and *R. chacei* with a mixotrophic diet combining symbiosis, bacterivory, and scavenging, to investigate whether links between diet and reproduction are also present in chemosynthetic ecosystems. The distribution of these species in two different hemispheres (Northern Atlantic for *R. exoculata* and *R. chacei*, Southern Indian Ocean for *R. kairei*) with similar levels of, but seasonally opposed, surface production allows us to evaluate whether their brooding periods are in synchrony with patterns of surface primary productivity.

## 2 MATERIALS AND METHODS

### 2.1 Field sampling

**FIGURE 1:**
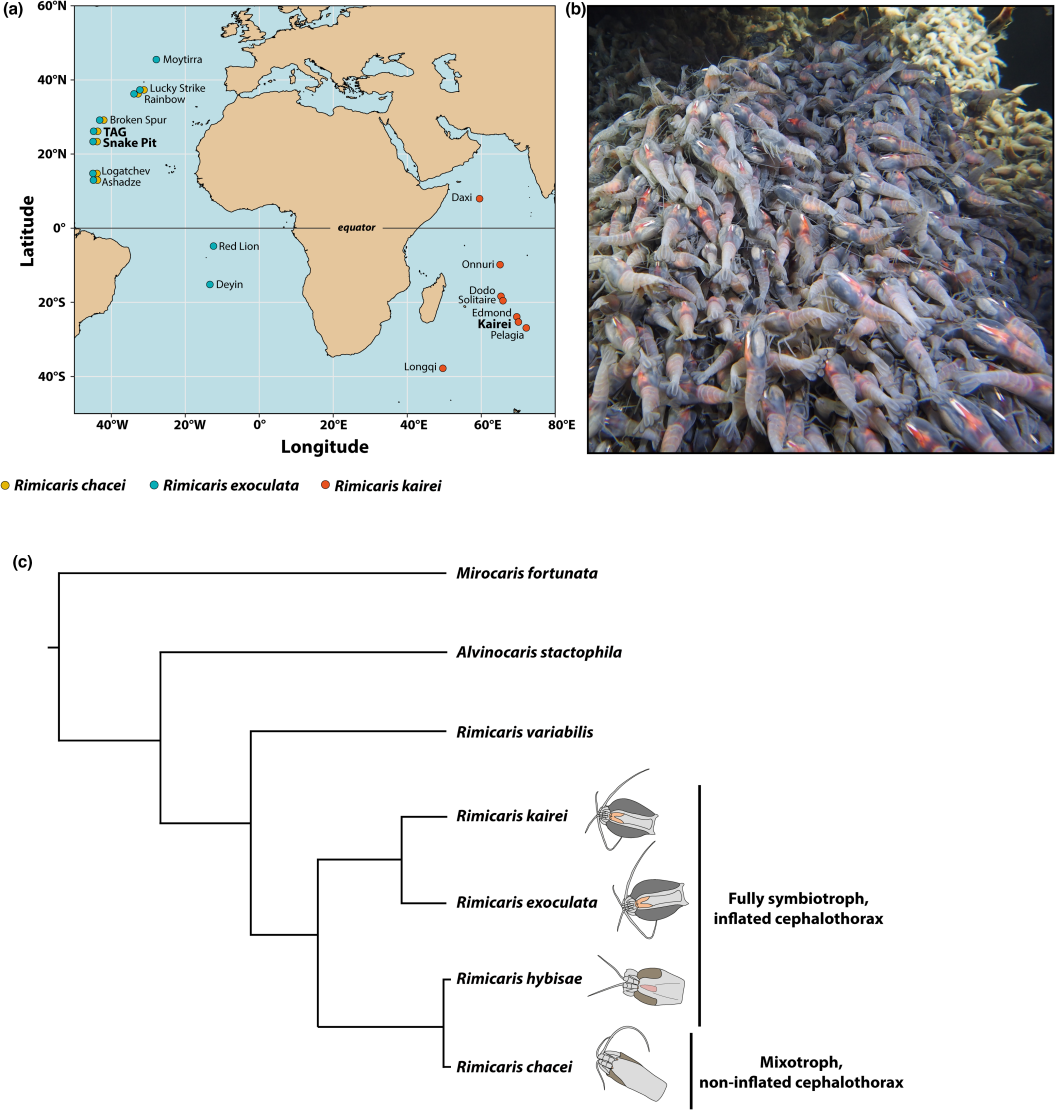
Context of the study. (a) Current geographic distribution of the three *Rimicaris* species studied with collection sites of this study highlighted in bold (b). Dense aggregations of *R. kairei* shrimps in February 2016 at the Kairei hydrothermal vent field. (c) Simplified schematic of the phylogenetic relationship of alvinocaridid shrimps discussed in the present study, based on Vereshchaka et al. (2015)

*Rimicaris exoculata* and *Rimicaris chacei* were collected from two hydrothermal vent fields (Figure 1a): TAG (26°08′12.12″N, 44°49′36.12″W, 3620 m depth) and Snake Pit (23°22′5.88″N 44°57′0″W, 3470 m depth) on the Mid‐Atlantic Ridge (MAR) between March and April in 2017 (HERMINE [https://doi.org/10.17600/17000200]) and between February and March in 2018 (BICOSE2 [https://doi.org/10.17600/18000004]). *Rimicaris kairei* were collected at one vent field (Figure 1a,b), Kairei (25°19′10.2″S, 70°2′24″E, 2415 m depth), on the Central Indian Ridge (CIR), between February and March in 2002 (expedition YK01‐15) and 2016 (expedition YK16‐E02), and in November 2009 (expedition YK09‐13). Shrimps were sampled using suction samplers of the human‐occupied vehicle (HOV) *Nautile* for the MAR sites and the HOV *Shinkai 6500* for the CIR. Upon recovery onboard, ovigerous females of *R. exoculata* and *R. chacei* were identified, individually sorted from the rest of the population, and fixed in 10% neutral buffered formalin for 24 h, before being rinsed and stored in 80% ethanol. For *R. kairei*, ovigerous females were identified and sorted in the laboratory after the cruise.

Ovigerous females included both brooding females and females that had just released their larvae but still retained modified pleopods with pieces of egg envelopes attached, characteristic of a recent brooding status (which we call “hatched broods” hereafter), following Nye et al. (2013). Ready‐to‐hatch broods were identified similarly, although these still had a few eggs left attached to their pleopods with clear fully developed larval structures. Broods consisted of fertilized eggs (hereafter sometimes called “eggs” for ease of language) containing developing embryos within an envelope, maintained together between the mother's modified pleopods. Within a brood, all embryos exhibited similar development stage. Embryonic stages in broods were identified and classified in three developmental stages (early, mid, and late) as seen through their transparent envelope. Briefly, early stage includes freshly fertilized eggs until the blastula stage, mid‐stage starts with gastrulation and extends to the end of the naupliar development (embryonic structures clearly separated from yolk), and late stage includes post‐naupliar development when abdomen, appendages, and larval structures become fully developed (Hernandez‐Avila et al., 2021; Methou et al., 2019).

### 2.2 Shrimp and egg measurements

Carapace length (CL) of each female was measured with Vernier calipers from the posterior margin of the ocular shield (or eye socket) to midpoint of the posterior margin of the carapace, with an estimated precision of 0.1 mm. For each brood, the total number of eggs was manually counted. Ten eggs were selected randomly to measure maximum and minimum egg diameters. The volume of eggs was estimated following the same method as Hernández‐Ávila et al. (2021), considering a spheroid volume $v=\frac{4}{3}\pi .r_{1}.r_{2}$, where r_1_ and r_2_ are half of maximum and minimum axis of each egg, respectively. Some ovigerous females harbored damaged broods where a part of the eggs was obviously missing while the remaining ones were clearly not ready to hatch, precluding natural spawning. These losses may be due to developmental abortion but most probably to sampling damages, and such broods were thus discarded from our fecundity analysis (17% of *R. exoculata*, 21.1% of *R. chacei*, and 7.1% of *R. kairei* broods collected). In addition, although the brooding state of females from the YK01‐15 (2002) expedition could be assessed, poor storage condition of these samples did not allow egg count or measurement analyses. An additional published dataset for *Rimicaris exoculata* collected at TAG and Snake Pit in January–February 2014 (BICOSE cruise) is also included in our analyses (Hernández‐Ávila et al., 2021), the raw data of which are available in the Ifremer/SEANOE (SEA scieNtific Open data Edition) database at: https://doi.org/10.17882/84112. As the 2014 dataset was produced by a different observer than the present study, a subsample of 60 broods from 2014 were measured again by the observer who produced the 2017 and 2018 datasets, and compared with the 2014 dataset produced by Hernández‐Ávila et al. (2021). The remaining broods (49) from the 2014 dataset were unavailable for remeasurement.

We expended a literature survey on the occurrence of *R. exoculata* brooding females at MAR vent sites initially conducted in 2016 Hernández‐Ávila et al. (2021) through the addition of the results of the present study and literature data available for the two other *Rimicaris* species included here. We also completed this information using video surveys conducted during several cruises at MAR (Serpentine 2007, Biobaz 2013, BICOSE 2014, Hermine 2017, BICOSE2 2018) and CIR vent sites (YK09‐13, YK16‐E02). A total of 270 h 46 min of video recording were qualitatively screened to evaluate the presence of brooding females in the shrimp assemblages. Brooding females could be identified in close‐view frames by the orange‐pink coloration of the ventral part of their abdomen indicative of the presence of a brood. This allows us to gather information on the presence of brooding females at different months of the year, and different vent sites of our study areas, over the last 35 years.

Proportions of ovigerous females within samples were calculated against the number of sexually mature females (see Appendix S1).

### 2.3 Statistical analysis

Brooding females were grouped according to sampling year, species, and vent field. Visual examination of our dataset and Shapiro–Wilk normality tests revealed that size, egg number, and egg volume of *Rimicaris* brooding females deviated from a normal distribution. Therefore, nonparametric tests were used for intergroup comparison, with a Kruskal–Wallis test followed by post hoc Dunn's tests when three or more groups were compared (see Appendix S1 for detailed *p*‐values of these tests in Tables S1 and S2). Spearman's rank–order correlation was used to assess relationship between log_*e*_‐transformed realized fecundity and log_*e*_‐transformed carapace length of the different shrimp groups. Egg stage proportions between groups were compared using the chi‐squared test with Yate's correction. All analyses were performed in R v. 4.0.3 (R Core Team, 2020).

## 3 RESULTS

### 3.1 Interannual variation of *R. exoculata* reproduction

**FIGURE 2:**
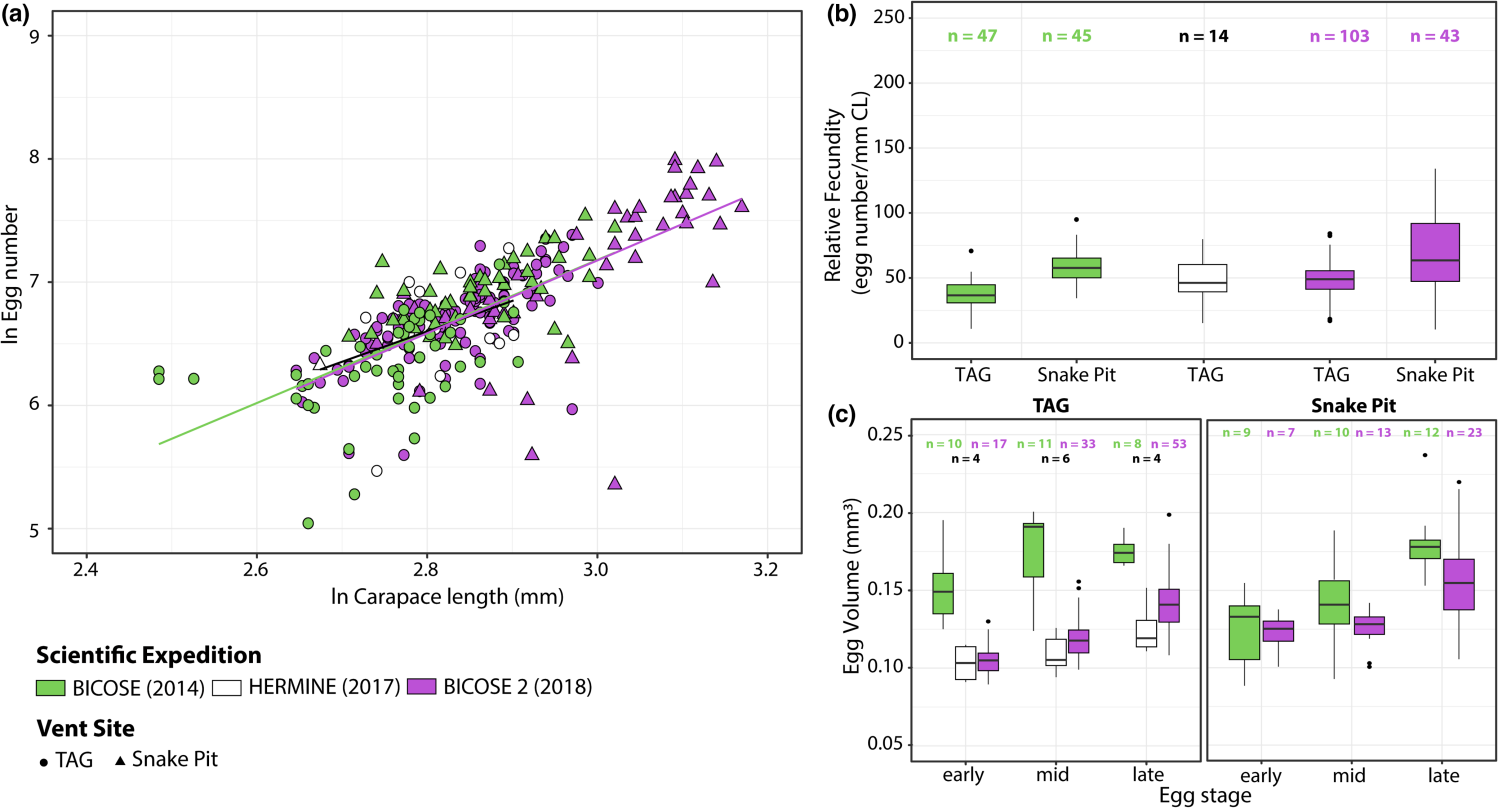
Interannual comparison of reproductive features of *Rimicaris exoculata* collected between 2014 and 2018. *n*: number of individuals for each condition. (a) Variation of log_e_‐transformed minimum realized fecundity with log_e_‐transformed carapace length. (b) Fecundity corrected for body size (carapace length). (c) Egg volumes at different stages

Realized fecundity of *Rimicaris exoculata* correlated positively with carapace length in both 2014 (Spearman's correlation *r* = .67, *p* < .0001) and 2018 (Spearman's correlation *r* = .73, *p* < .0001) (Figure 2a). Moreover, slopes of the relationship between log_*e*_‐transformed realized fecundity and log_*e*_‐transformed carapace length did not differ significantly between these two sampling years (*F* = 1.175, *p* = .28) or between TAG and Snake Pit in 2018 (*F* = 0.004, *p* = .95). Although no correlation was found in 2017 (Spearman's correlation *r* = .38, *p* = .16), probably due to the lower sampling size, realized fecundity in 2017 still fell within the range of the other sampling years (Figure 2a).

Overall, size‐specific fecundity ranged from 19 to 95 eggs mm^−1^ in 2014, from 15 to 80 eggs mm^−1^ in 2017, and from 10 to 134 eggs mm^−1^ in 2018 (Figure 2b). Limited differences in size‐specific fecundities were observed between different sampling years (Kruskal–Wallis test, *H* = 65.025, *p* < .05) with significant variations between 2014 and 2018 at TAG only (2014: 37.3 ± 11.4; 2018: 49.4 ± 12.9; Dunn's multiple comparison test, *p* < .05). Differences in size‐specific fecundity were also observed between the two vent fields both in 2014 and in 2018, (Dunn's multiple comparison tests, *p* < .05). Carapace length of brooding females from TAG was indeed larger in 2018 than in 2014 (Dunn's multiple comparison test, *p* < .05), suggesting that size‐specific fecundity increase was attributable to this larger body size in 2018, indicating that an exponential relationship likely exists between body size and fecundity. In addition, carapace lengths of brooding females were always larger at Snake Pit than at TAG both in 2014 and in 2018 (Dunn's multiple comparison tests, *p* < .05).

To avoid potential observer bias in the measure of egg volumes, a subsample of eggs from 2014 were remeasured by the same observer who produced the 2017 and 2018 datasets and compared with the 2014 dataset produced by Hernández‐Ávila et al. (2021) (Figure S2). This uncovered a significant observer effect for eggs from Snake Pit with significantly larger egg volumes for the broods observed by Hernández‐Ávila et al. (2021) (Kruskal–Wallis test, *H* = 11.849, *p* < .05) but not for TAG, with similar egg volumes between the two observers (Kruskal–Wallis test, *H* = 0.91471, *p* = .33). Due to this, we discarded the egg volume measurements made by Hernández‐Ávila et al. (2021) and used only data measured by the same observer herein for analyses, which revealed variations in egg volumes among the different sampling years (Kruskal–Wallis test, *H* = 60.897, *p* < .05) (Figure 2c). At TAG, egg volumes were found to be larger in 2014 than in other years of sampling across all developmental stages (Dunn's multiple comparison tests, *p* < .05), whereas the egg volume was only larger in 2014 (Dunn's multiple comparison test, *p =* .019) for late developmental stages at Snake Pit.

Proportions of each developmental stage were rather similar between 2014 and 2017 (TAG: *χ* ^2^ = 0.546, 2 *df*, *p* > .05) with a majority of mid‐stage embryos in broods (2014: 48.9%; 2017: 42.9%) (Figure S3a). In contrast, these proportions differed between 2014 and 2018 at both vent fields (TAG: *χ* ^2^ = 27.938, 2 *df*, *p* < .05; Snake Pit: *χ* ^2^ = 58.493, 2 *df*, *p* < .05) as most embryos from 2018 broods were at a late stage (TAG: 51.5%; Snake Pit: 53.5%) (Figure S3a).

### 3.2 Variations in reproductive patterns between *Rimicaris* species

**FIGURE 3:**
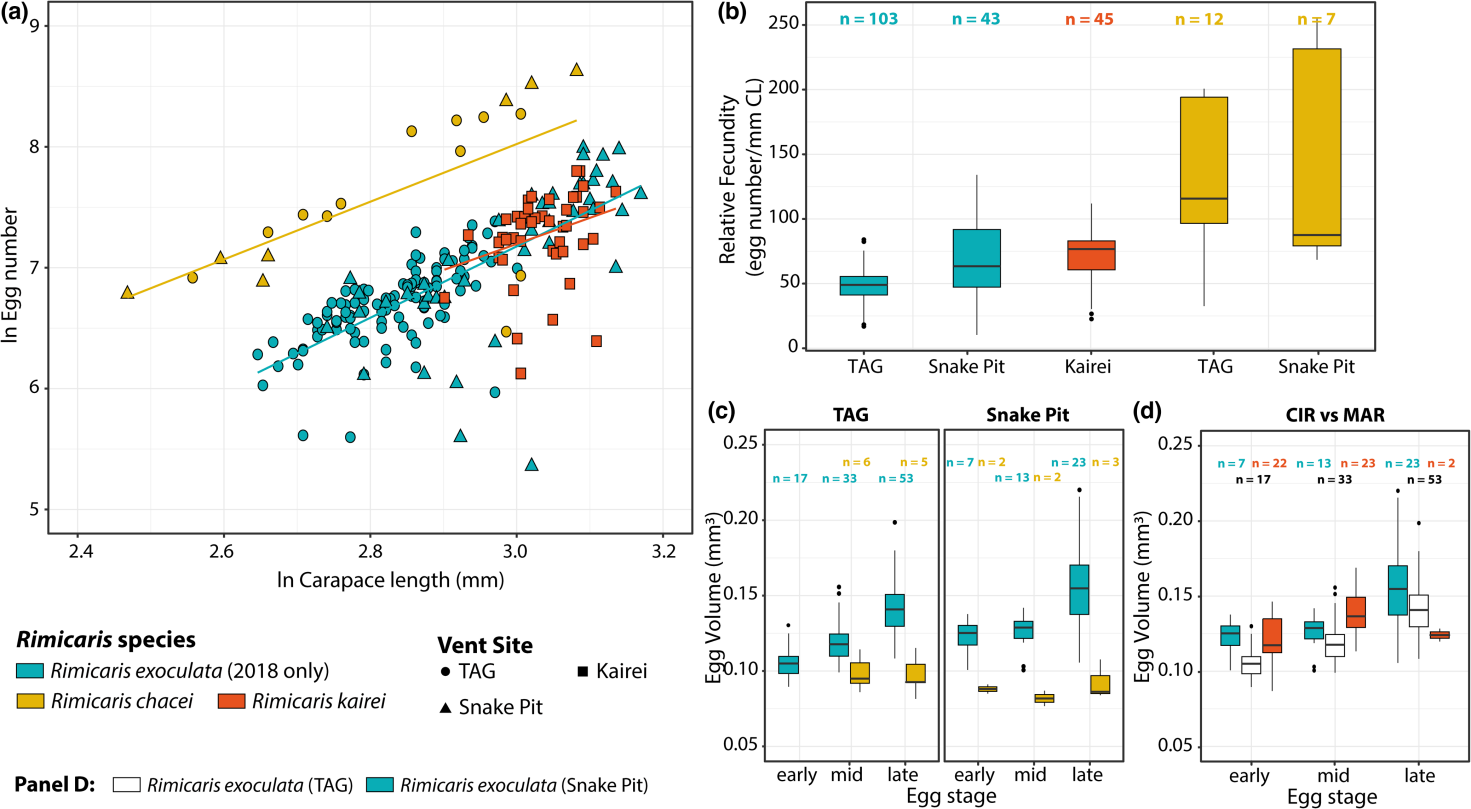
Comparison of reproductive features between *Rimicaris* species from the MAR and the CIR. *n*: number of individuals for each condition. (a) Variation of log_e_‐transformed minimum realized fecundity with log_e_‐transformed carapace length. (b) Fecundity corrected for body size (carapace length). (c) Egg volumes at different stages of *Rimicaris* shrimps from the MAR. (d) Egg volumes at different stages of *R. exoculata* and *R. kairei*

Like *R. exoculata*, the realized fecundity of *R. kairei* and *R. chacei* correlated positively with carapace length (Spearman's correlation: *R. kairei*: *r* = .40, *p* = .0044; *R. chacei*: *r* = .70, *p* = .00066) (Figure 3a). Moreover, the slopes of the relationship between log_*e*_‐transformed realized fecundity and log_*e*_‐transformed carapace length did not significantly differ between *R. exoculata* and its sister species *R. kairei* (*F* = 1.6168, *p* = .2054). However, they significantly differed between *R. exoculata* and its co‐occurring congener *R. chacei* (*F* = 6.6376, *p* < .05).

Overall, size‐specific fecundity ranged from 23 to 112 eggs mm^−1^ for *R. kairei* and from 26 to 255 eggs mm^−1^ for *R. chacei* (Figure 3b). Large differences in size‐specific fecundity were observed between *R. chacei* and other *Rimicaris* species (Kruskal–Wallis test, *H* = 68.836, *p* < .05). Size‐specific fecundity of *R. chacei* was always significantly greater than *R. exoculata* both at TAG (*R. exoculata*: 49 ± 13; *R. chacei*: 129 ± 59; Dunn's multiple comparison test, *p* < .05) and at Snake Pit (*R. exoculata*: 69 ± 31; *R. chacei*: 148 ± 87; Dunn's multiple comparison test, *p* < .05). These differences were independent of size variations between the two species with *R. chacei* brooding females being either of similar size at TAG (Dunn's multiple comparison test, *p* > .05) or even smaller at Snake Pit (Dunn's multiple comparison test, *p* < .05). Smaller differences in size‐specific fecundities were observed between *R. exoculata* and *R. kairei* with only a higher size‐specific fecundity for *R. kairei* (72 ± 20) from Kairei compared with *R. exoculata* from TAG (49 ± 13; Dunn's multiple comparison test, *p* < .05). Such variations may be linked to differences in body size of brooding females, as *R. exoculata* from TAG were smaller on average than *R. kairei* from Kairei (Dunn's multiple comparison test, *p* < .05).

Large differences were also observed in egg volumes between the three *Rimicaris* species (Kruskal–Wallis test, *H* = 49.80, *p* < .05) (Figure 3c,d). Thus, egg volumes of *R. chacei* were significantly smaller than those of *R. exoculata* at any stage across all vents (Dunn's multiple comparison test, *p* < .05) (Figure 3c). In contrast, differences in egg volumes were more limited between *R. exoculata* and *R. kairei* with significantly larger eggs for *R. kairei* from Kairei compared with *R. exoculata* from TAG for eggs with mid‐stage embryos only (Figure 3d). Surprisingly, we did not find gradual increase in egg volumes between successive developmental stages of *R. chacei* as was observed in the two other *Rimicaris* species, either at TAG (Kruskal–Wallis test, *H* = 2.57, *p* > .05) or at Snake Pit (Kruskal–Wallis test, *H* = 0.61, *p* > .05). Of note, we did not observe ready‐to‐hatch or hatched egg broods in *R. chacei* as was seen in the other shrimps.

In addition, proportions of each embryonic stage were relatively similar between *R. exoculata* and *R. chacei* broods at both sites (TAG: *χ* ^2^ = 84.837, 2 *df*, *p* < .05; Snake Pit: *χ* ^2^ = 84.837, 2 *df*, *p* < .05) with the majority harboring late‐stage embryos (Figure S3b). On the contrary, *R. kairei* broods from Kairei were mostly with early‐ or mid‐stage embryos (early: 46.8%; mid: 48.9%).

### 3.3 Temporal variations in the presence of *Rimicaris* ovigerous females

**FIGURE 4:**
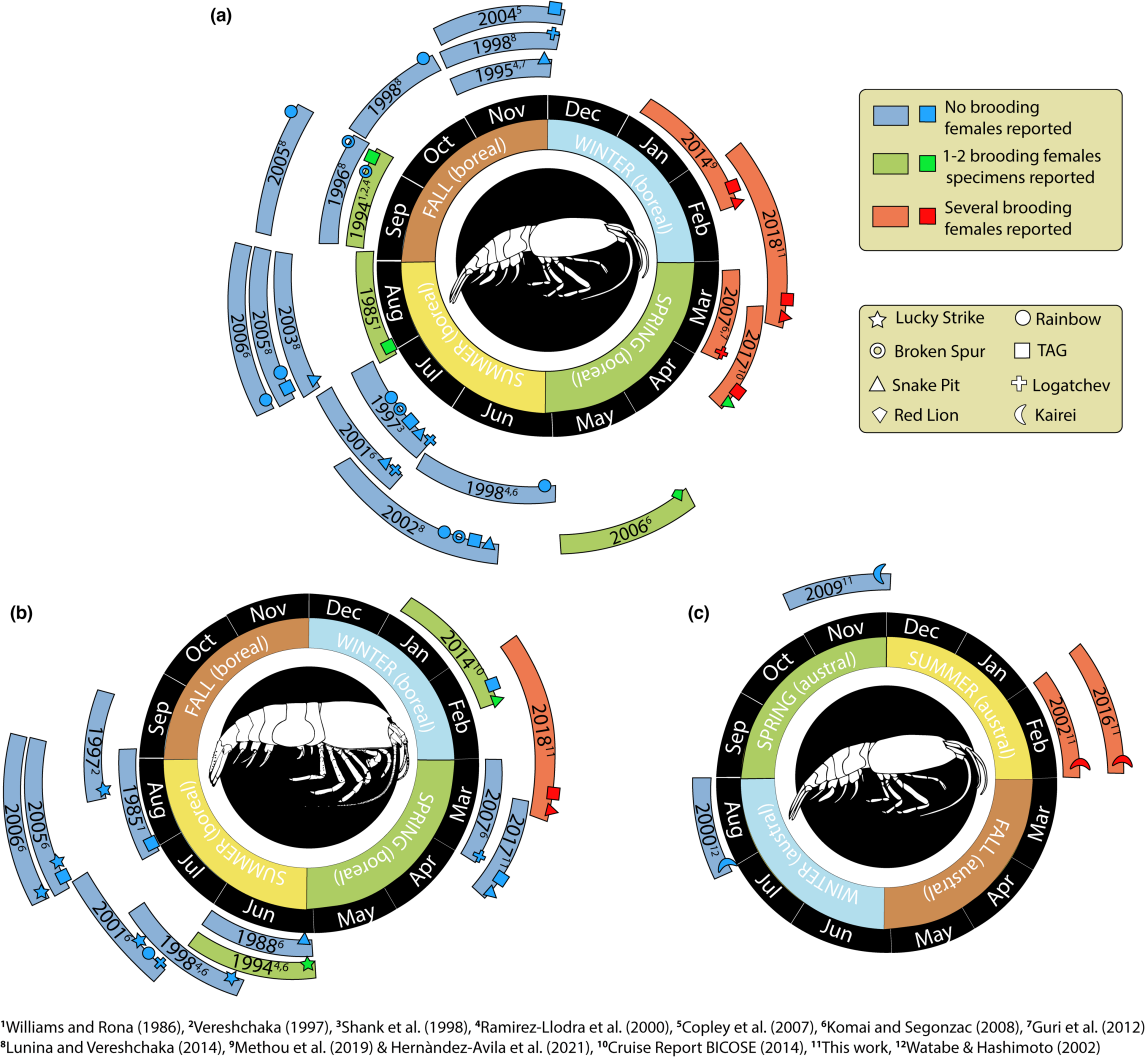
Diagrams summarizing the occurrence of reproductive stages of the studied *Rimicaris* shrimp species over different samplings realized between 1985 and 2018 according to the year period (a) for *R. exoculata*, (b) for *R. chacei*, and (c) for *R. kairei*

A large number of ovigerous females of *R. exoculata* and *R. chacei* were retrieved in February 2018 at both MAR vent fields (Figure 4a). Indeed, ovigerous females represented 55.4% and 18.2% of all the sexually mature *R. exoculata* females collected respectively at TAG and Snake Pit and 70.6% or 32.3% of all the sexually mature *R. chacei* females, respectively, at TAG and Snake Pit. Ovigerous females of *R. exoculata* were also present between late March and April 2017 although in a relatively lower proportion, constituting 25.7% and 4% of sexually mature females collected at TAG and Snake Pit, respectively (Figure 4a). The majority of *R. exoculata* ovigerous females were collected in dense aggregations close to vent orifices with only two ovigerous females collected outside these assemblages. On the contrary, ovigerous females of *R. chacei* were collected either in visually “hidden” aggregations under mussels beds or sulfide block interstices where large populations of their adults have been retrieved (Methou et al., 2022), or within dense aggregations of *R. exoculata*. Substantial spatial variations were observed in the proportions of *R. exoculata* ovigerous females among the different densely aggregated shrimp assemblages. Ovigerous females comprised between 5.6% and 69.6% of the sexually mature females for those collected in 2018 (Figure S4) and between 3.6% and 21.6% from assemblages collected in 2017 (Figure S5). Additionally, ovigerous females were absent in four of the seven dense aggregates collected in 2017 but only in 3 of the 15 dense aggregates collected in 2018. As highlighted previously (Hernández‐Ávila et al., 2021), ovigerous females of *R. exoculata* were almost absent from samples collected outside this period of the year—that is, January to April—despite more than 35 years of sampling efforts along the MAR (Figure 4a). Similarly, ovigerous females of *R. chacei* were almost absent from other sampling reports, with only one ovigerous females in June 1994 at Lucky Strike (Figure 4b). Spatial variations in the proportions of *R. chacei* ovigerous females among sampling events were between 46.2% and 73.3% of sexually mature females collected in February 2018 (Figure S4). These reproductive stages were present at three of the four populations of *R. chacei* investigated, where the number of adults collected was greater than five individuals.

Large numbers of ovigerous *R. kairei* were also retrieved in February 2002 and 2016 at Kairei on the CIR (Figure 4c), constituting respectively 16.3% and 19.2% of sexually mature females collected at this vent field. These reproductive stages were present in every dense aggregations of *R. kairei*, with spatial variations between sampled aggregations being 3.4% and 24.1% of all the sexually mature females collected in February 2016 (Figure S6). Conversely, ovigerous females were completely absent from samples collected in November 2009 (Figure 4c). Like *R. exoculata*, all of these ovigerous females were collected in dense aggregations of shrimps next to hot fluid emissions.

## 4 DISCUSSION

Altogether, our results show that *Rimicaris exoculata* and *R. kairei* had much lower size‐specific fecundities and much larger egg volumes than *R. chacei*. The realized fecundity correlated positively with carapace length in all three species, although the relationship between the two variables was distinct in *R. chacei* compared with the two other species. Spawning appears to be seasonal in all three species, occurring between January and April, possibly cued to optimal larval release conditions. Surprisingly, the data we present suggest a similar spawning season for *R. kairei* and the other two species, despite their experience of opposite primary production trends as they were collected at opposing latitudes in two different hemispheres.

### 4.1 Distinct reproductive strategies in *Rimicaris* shrimps with evidence of diet influence

*Rimicaris exoculata* and *Rimicaris chacei* exhibit distinct reproductive traits unlikely to simply result from differences in mean body sizes. *Rimicaris kairei* is similar to *R. exoculata*, in terms of size‐specific fecundity and mean egg size. Our results indicate that the two groups (*R. exoculata* and *R. kairei*, vs *R. chacei*) are situated at two ends of the trade‐off between size‐specific fecundity and egg sizes currently observed within alvinocaridids (Copley & Young, 2006; Nye et al., 2013; Ramirez‐Llodra et al., 2000; Ramirez‐Llodra & Segonzac, 2006). *Rimicaris chacei* brooding females display one of the highest size‐specific fecundities and one of the smallest mean egg volumes among alvinocaridids, whereas *R. exoculata* and *R. kairei* brooding females present a rather low size‐specific fecundity and a large mean egg volume for this family. In addition, annual comparison of *R. exoculata* reproductive traits also revealed a stable size‐specific fecundity over the years, but some levels of variation were seen in the mean egg volume among the sampling years.

These variations between species mirror differences observed in benthic post‐settlement stages of these two shrimp groups, with *R. chacei* exhibiting significantly smaller body size for settled juveniles, as well as much larger relative proportions of juveniles compared with adults in populations (Methou et al., 2020, 2022). These previous works on the population demographics of the two MAR species showed a drastic post‐recruitment collapse in *R. chacei* with a high juvenile mortality, hypothesized to be the result of a combination of factors, including interspecific competition between juveniles of the two congeneric species and size‐specific predation of the surrounding fauna on *R. chacei* juveniles (Methou et al., 2022). Such a collapse was not observed in *R. exoculata* populations, which exhibited a larger proportion of adults than juveniles, potentially close to the carrying capacities of their vent field. These differences in terms of age‐ and size‐specific selection acting heterogeneously on the different life stages of the two species could be the source of their niche partitioning (Methou et al., 2020, 2022) and lead to the different life history strategies observed herein for these species. Furthermore, density‐dependent selection could be a driving factor, where *R. exoculata* is always nearing the equilibrium population, and therefore invest more in each offspring (Methou et al., 2020, 2022), while *R. chacei* has less stable populations and therefore produces more offsprings.

The integration in this theoretical framework of *R. hybisae*, the sister species of *R. chacei* from vents on the Mid Cayman Spreading Centre, phylogenetically close but behaviorally distinct, is interesting as size‐specific fecundity of *R. hybisae* is more similar to that of *R. exoculata* and *R. kairei* rather than *R. chacei* (Nye et al., 2013). *Rimicaris hybisae* share with *R. exoculata/R. kairei* both a comparable ecological context, living in dense aggregations close to vent fluids emissions, and a comparable gross morphology, with an enlarged cephalothorax (Zbinden & Cambon‐Bonavita, 2020), characters lacking in *R. chacei*. These are linked to distinct feeding strategies between the two groups of shrimps, with a mixotrophic diet for *R. chacei* (Apremont et al., 2018; Gebruk et al., 2000; Methou et al., 2020) and dependency on chemosynthetic symbionts for others including *R. hybisae* (Gebruk et al., 2000; Methou et al., 2020; Rieley et al., 1999; Streit et al., 2015; Van Dover, 2002). Similar to *R. exoculata* and *R. kairei*, *R. hybisae* is also extremely abundant where it occurs and the adult population is potentially nearing the carrying capacity. We therefore posit that the differences we observe between fecundities in our three *Rimicaris* shrimps are likely linked to their feeding ecology (and occupied niche), rather than being constrained by their phylogeny.

Despite variations in average egg number, mean egg sizes of *R. hybisae* and *R. chacei* were in contrary comparable with similar maximum and minimum egg diameters (Nye et al., 2013). Such similarities on mean egg sizes were more unexpected and would deserve further work, including additional alvinocaridid shrimp species with different feeding strategies to elucidate whether egg sizes could be influenced by feeding strategies or by other factors such as phylogeny. A potential observer effect, as shown here for egg volumes of *R. exoculata* collected during the BICOSE expedition, cannot be excluded either.

### 4.2 The unusual reproductive cycles of *Rimicaris* shrimps

We report here a much‐increased presence of ovigerous *R. exoculata* in February 2018 and to a lower extent in late March–April 2017, compared with other periods of the year. Combined with previous data from the literature (summarized in Figure 4a), these results support the existence of a brooding period for *R. exoculata* mostly during boreal winter, starting in January–February and ending around March–April. Moreover, we did not observe significant variability in reproductive features of *R. exoculata*, either in terms of fecundity or proportions of reproductive females, between different sampling years—indicating a relative interannual stability of these traits (Figure 2). Since gamete production can be sustained continuously in species depending on local chemosynthesis for their diet, mechanisms driving periodicity in shrimp life cycles are more likely to act on their planktotrophic larval phase through seasonality in availability of photosynthetically derived food (Eckelbarger and Watling, 1995). The few previous studies looking at a limited number of samples indeed reported asynchronous ovarian development for *R. exoculata*, taken to suggest aperiodic egg production (Copley et al., 2007; Ramirez‐Llodra et al., 2000). However, the maximum oocyte size appeared to be lower in individuals collected in summer than in those collected in autumn, regardless of the vent site (Hernández‐Ávila et al., 2021; Ramirez‐Llodra et al., 2000). Thus, this may result from a gradual size increase in mature oocytes in ovaries, culminating in winter, prior to spawning. Furthermore, oogenesis in *Rimicaris* might also be distinct from spawning activity, with mature ovaries retained for extended periods until the seasonal spawning, as observed in some deep‐sea echinoderms (Eckelbarger & Watling, 1995), followed by brooding and larval hatching.

Large proportions of *R. chacei* ovigerous females were also retrieved in February 2018 at the two vent fields, in contrast to historical sampling at other periods along the MAR (Figure 4b). Hence, a similar periodicity in reproduction could exist for both *Rimicaris* shrimps at MAR. Ovigerous *R. chacei* females were, however, scarce in other expeditions both during and outside this period (Figure 4b), which might be due to difficulties in sampling this rather fast swimming species, thus preventing so far, a full appreciation of its reproductive activity. A similar seasonal pattern was also suggested for the sister species of *R. chacei*, *R. hybisae*, although based on a limited dataset (Nye et al., 2013). Taken alone, these results on north MAR *Rimicaris* populations seemed to indicate aperiodic gametogenesis coupled with seasonal spawning, synchronized by the peak of photosynthetic production sinking from surface, as reported in other deep‐sea animals with seasonal reproductive periodicity (Dixon et al., 2006; Perovich et al., 2003; Tyler et al., 2007).

However, a different picture emerges when CIR *Rimicaris* populations are included as large proportions of *Rimicaris kairei* ovigerous females were found during austral summer in February 2002 and 2016, while they were absent from collections in August 2000 and November 2009 (Figure 4c). Spawning in *R. kairei* thus appears to follow the same timing as Atlantic *Rimicaris* species. This pattern was unexpected given their distribution in different hemispheres, at vent fields with opposite latitudes (Figure 1a) where these shrimps are expected to experience opposite trends in terms of surface primary production along the year (Harms et al., 2021; Pabortsava et al., 2017), driving opposite reproductive timing. Although our dataset for *R. kairei* is more limited than for *R. exoculata*, our results show that the two sister species share the same periodic brooding cycle, regardless of local seasonal variations in surface photosynthetic productivity, which also appear to be relatively weak in their respective regions (Harms et al., 2021; Pabortsava et al., 2017).

These observations cannot be generalized to the entire Alvinocarididae family, however, as evidences of potential aperiodic brooding have been published for some other species in the family. Regular sampling of *Mirocaris fortunata*, for example, yielded ovigerous females throughout the year (Komai & Segonzac, 2003; Methou, 2019; Ramirez‐Llodra et al., 2000) and similarly in the sampling records of *Rimicaris variabilis* (Komai & Tsuchida, 2015; Komai et al., 2016). It therefore appears unlikely that phylogenetic constraints could have maintained periodic brooding in some alvinocaridids but not in others (Figure 1c). Then again, some alvinocaridid species may also be influenced by seasonality in the surface production, like *Alvinocaris stactophila* that inhabit relatively shallow cold seeps under highly productive coastal waters and exhibit a brooding period between November and March (Copley and Young, 2006), matching the surface productivity. Hence, several mechanisms relating reproductive rhythms and environmental variability may coexist in vents and other deep‐sea ecosystems, even within the same family of animals.

Besides light and food supply, temperature has also been recognized as an important external timing cue in marine organisms (Mat, 2019) but observations from long‐term observatories deployed in a few vent fields did not reveal any seasonal variations in temperature at an annual scale (Barreyre et al., 2014; Cuvelier et al., 2017). Moreover, hydrothermal vent ecosystems, compared to most of the deep sea where temperature is relatively constant within regions, exhibit heat anomalies with steep and unpredictable gradients at small spatial scales even within the same animal aggregation (Schmidt et al., 2008). In the current state of our knowledge, the synchronizing factors driving reproductive cycles in *Rimicaris* shrimps from the MAR and the CIR remain unclear. Recently, it was revealed that bathymodiolin mussels inhabiting vents on the MAR retain and express circadian clock genes following tidal cycles, which may be mediated by either tidal stimulus or an internal clock (Mat et al., 2020). It is not completely out of the question that the *Rimicaris* shrimp species studied herein may also use a set of internal biological clocks to time their reproductive activity.

Overall, even if primary surface production may be a key synchronizing cue for some deep‐sea species, as demonstrated in bathymodiolin mussels and vent crabs (Dixon et al., 2006; Tyler et al., 2007), our results underscore that reproductive cycles and seasonality in the deep sea are not as simple as it was once thought to be. A thorough understanding of the reproductive ecology of the dominant species shaping the ecosystem is key to assessing the resilience of deep‐sea ecosystems, but remain incomplete so far even for emblematic taxa such as *Rimicaris*. With deep‐sea mining for massive sulfides and other resources being imminent (Van Dover et al., 2018), the habitat of these unique animals that can live nowhere else is being threatened. Research expeditions in a particular region tend to be conducted in similar timings of the year due to seasonal variations in sea and weather conditions, but our results show that communities need to be revisited at different times of the year. Elucidating the ecology of these species is now more important than ever, and our data exemplify the importance of time‐series studies.

## AUTHOR CONTRIBUTIONS

**Pierre Methou:** Conceptualization (equal); data curation (lead); formal analysis (lead); writing – original draft (lead); writing – review and editing (equal). **Chong Chen:** Resources (equal); writing – original draft (equal); writing – review and editing (equal). **Hiromi Kayama Watanabe:** Resources (equal); writing – review and editing (equal). **Marie‐Anne Cambon:** Funding acquisition (equal); resources (equal); supervision (equal); validation (equal); writing – review and editing (equal). **Florence Pradillon:** Conceptualization (equal); data curation (equal); resources (equal); supervision (lead); validation (lead); writing – original draft (equal); writing – review and editing (equal).

## CONFLICT OF INTEREST

The authors declare they have no conflict of interests.

## Supporting information


Appendix S1

## Data Availability

Dataset used within this manuscript can be accessed through the Ifremer SEANOE (SEA scieNtific Open data Edition) database at: https://doi.org/10.17882/84195.
