# Supplementary material for: Reproduction in deep‐sea vent shrimps is influenced by diet, with rhythms apparently unlinked to surface production
Source: Ecol Evol. 2022 Jul 17;12(7):e9076. doi: 10.1002/ece3.9076 (PMC9288886; doi:10.1002/ece3.9076)
Supplement: Supplementary file 1 — Appendix S1 [file ECE3-12-e9076-s001.pdf]

Materials & Methods

Estimation of the size of effective sexual maturity (ESM)

The size of effective sexual maturity (ESM) of *R. chacei* and *R. kairei* was estimated according to (Hernández-Ávila et al., 2021; King, 2007), from the proportion of ovigerous females per size class, corrected by the maximum proportion of ovigerous females.

The proportion of ovigerous female specimens were plotted against size classes and fitted to the logistic equation: Povf = $1/(1+e^{(a-b*CL)})$ where Povf is the proportion of ovigerous shrimp females, CL is the carapace length, and a and b are constants. The ESM was estimated by the size at which Povf is 50% (figure S1).

Results

Estimation of the size of effective Sexual Maturity (ESM)

We estimate the size at effective sexual maturity (ESM) at 12.65 mm for *R. chacei* females (figure 1a) and at 15.4 mm for *R. kairei* (figure 1b). Thus, females with size ≥ 12.65 mm for *R. chacei* and with size ≥ 15.4 mm for *R. kairei* were considered as sexually mature.

These ESM sizes correspond respectively to 58% and 65% of the maximal size reported for each species (*R. chacei*: 21.8 mm; *R. kairei*: 23.7 mm). Additionally, we reevaluate this proportion given previously for *R. exoculata* (Hernández-Ávila et al., 2021), which ESM represent 62% of the new maximal size reported for this shrimp species in our 2018 dataset (*R. exoculata*: 24.4 mm ;(Methou et al., 2021)).

Figure Caption

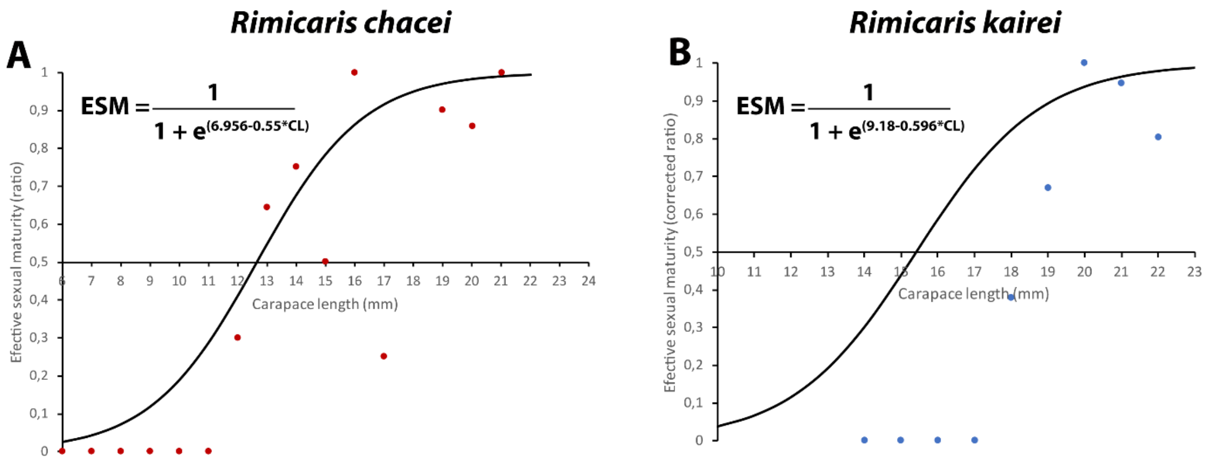

Figure S1. Estimation of effective sexual maturity (ESM) of **a.** *R. chacei* and **b.** *R. kairei*.

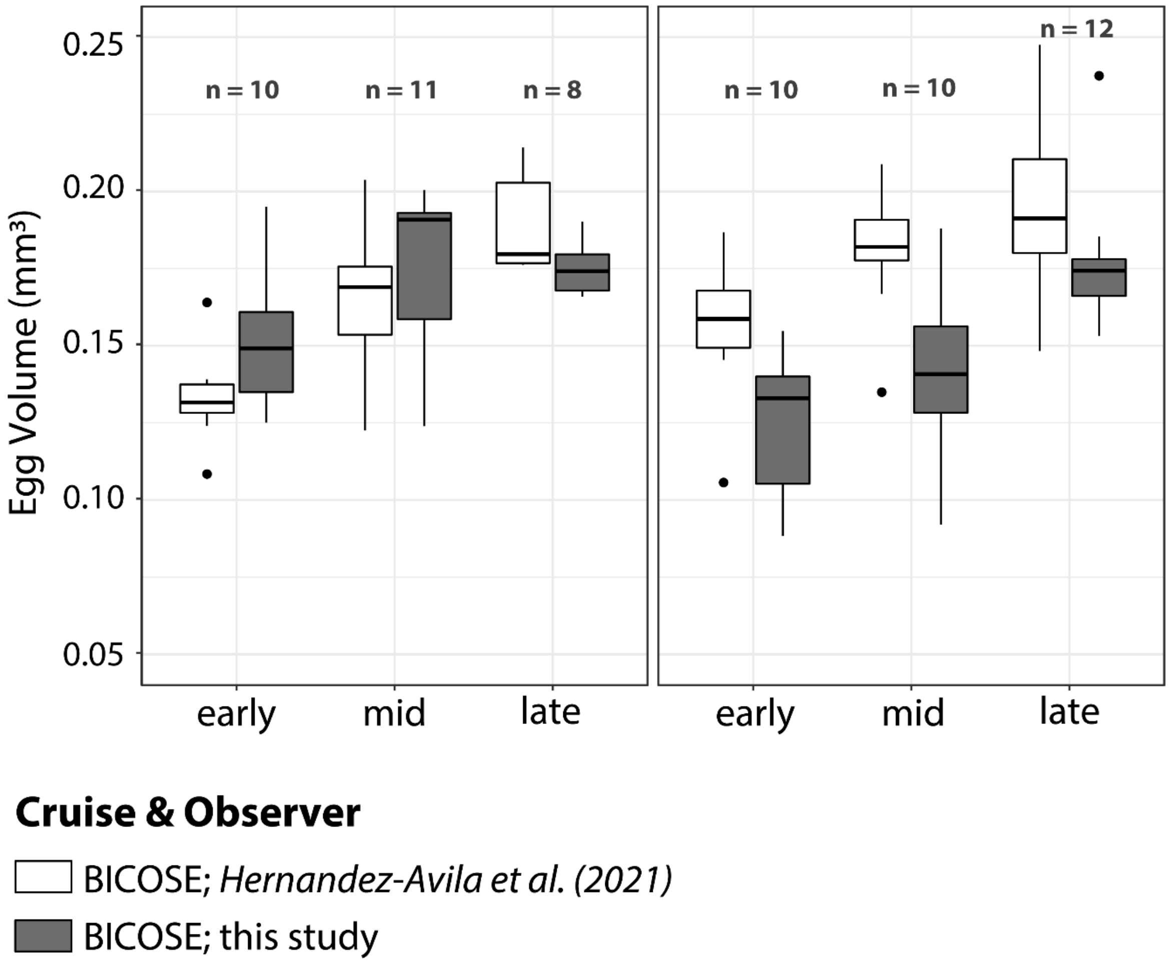

Figure S2. Evaluation of a potential observer effect in the comparison of egg volumes collected from different sampling expeditions.

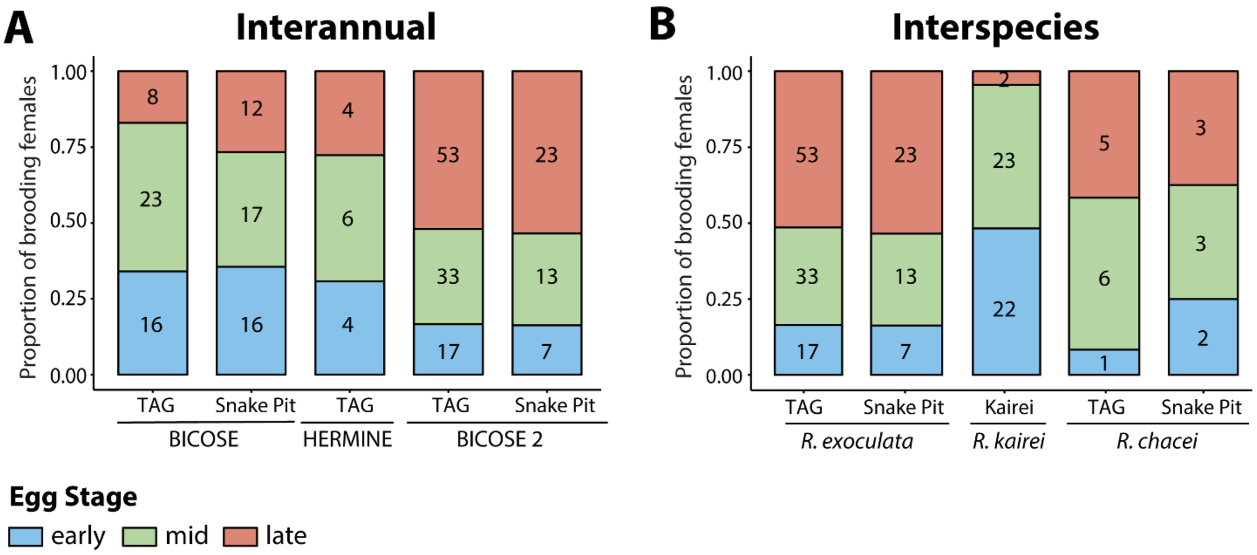

Figure S3. Proportions of each egg developmental stages. Numbers within the plots indicates the number of broods per developmental stages. **a.** Comparison of egg developmental stage proportions between *R. exoculata* broods from different sampling years. **b.** Comparison of egg developmental stage proportions between broods of the different *Rimicaris* species from the MAR and the CIR.

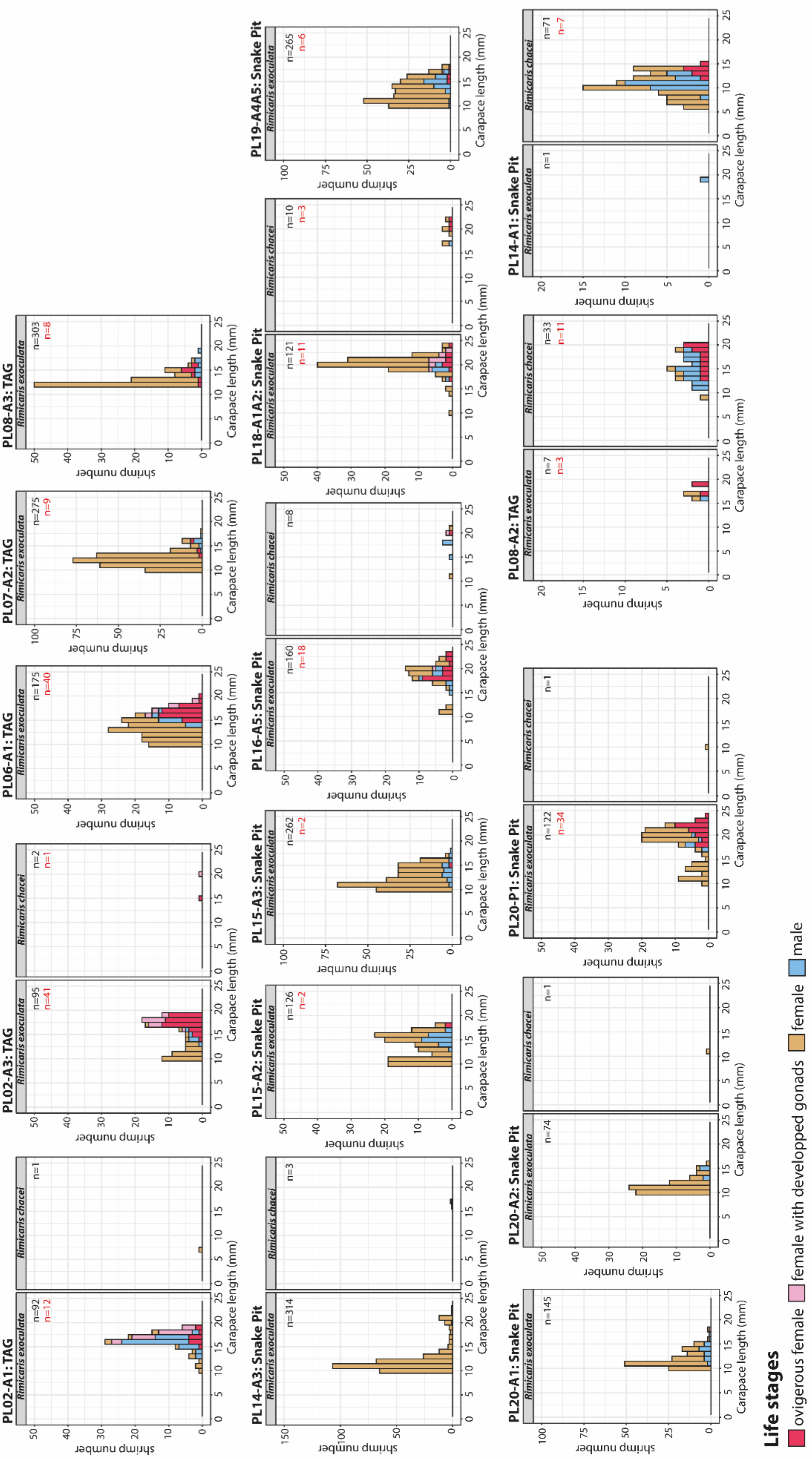

Figure S4. Small-scale spatial variations in size-frequency distribution of *Rimicaris* reproductive stages collected during the BICOSE 2 expedition in 2018. **n:** (black) number of measured adult individuals; (red) number of measured ovigerous females.

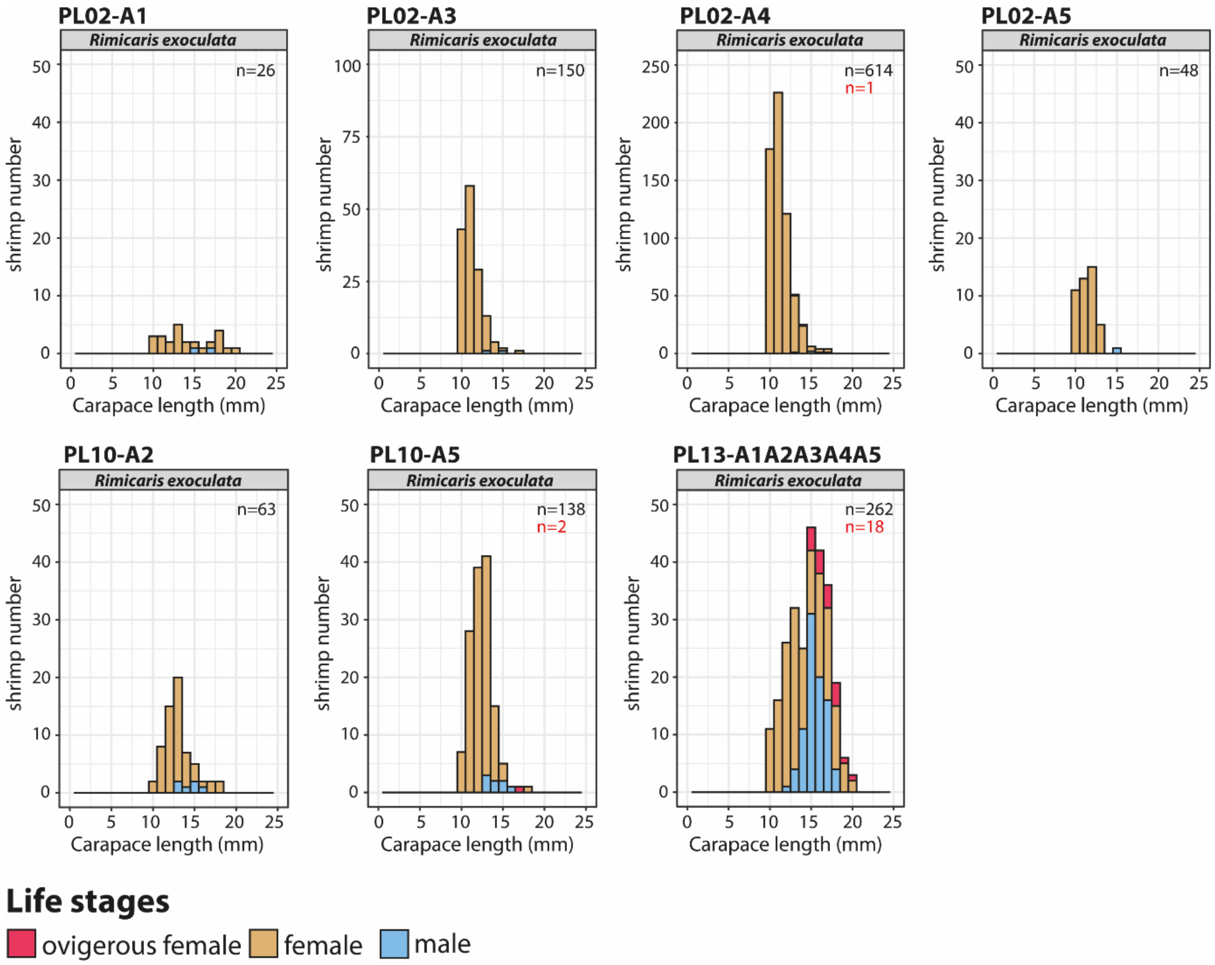

Figure S5. Small-scale spatial variations in size-frequency distribution of *R. exoculata* reproductive stages collected during the HERMINE expedition in 2017. **n:** (black) number of measured adult individuals; (red) number of measured ovigerous females.

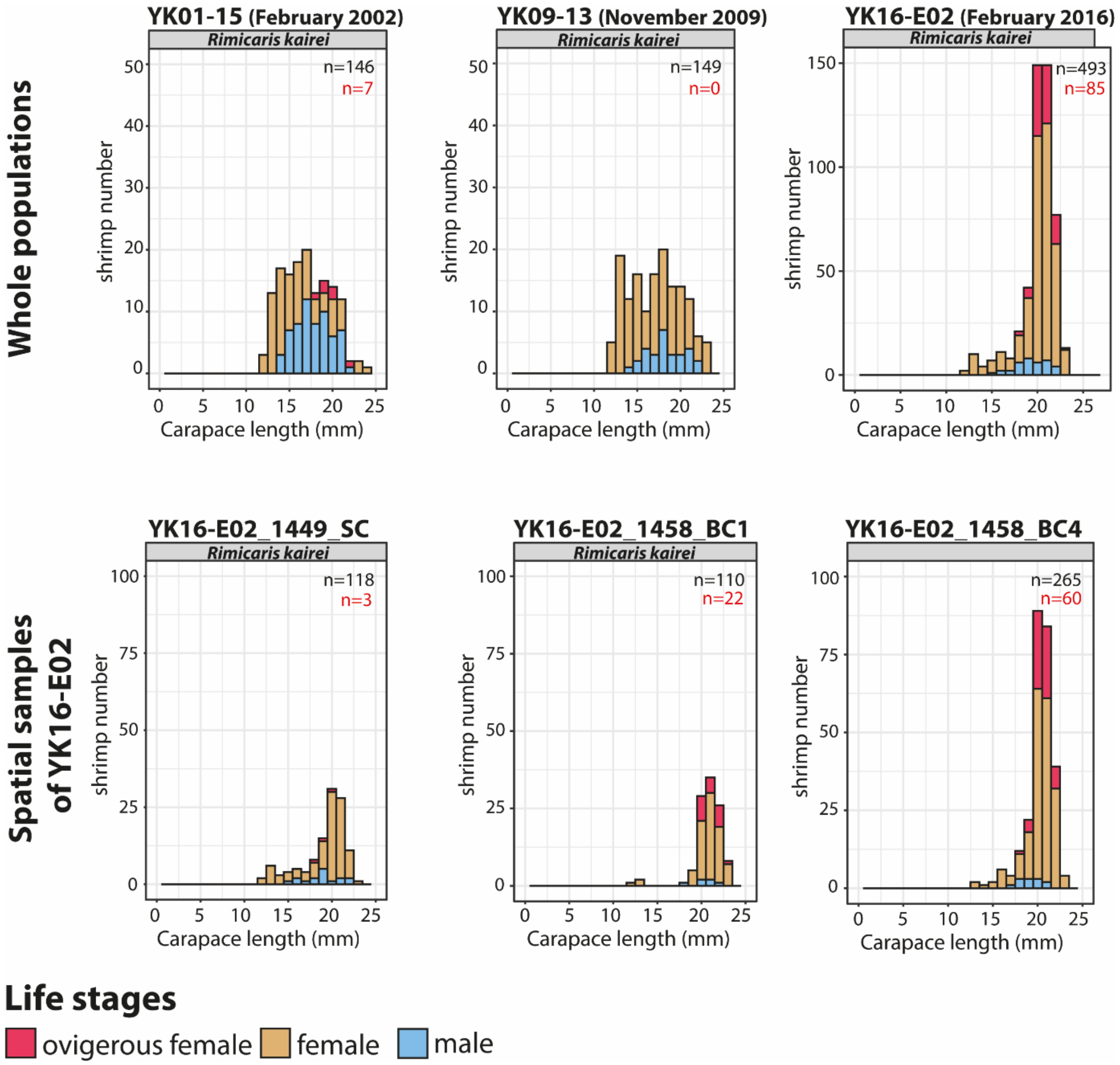

Figure S6. Small-scale spatial variations in size-frequency distribution of *R. kairei* reproductive stages collected during the YK16-E02 2 expedition in 2016. **n:** (black) number of measured adult individuals; (red) number of measured ovigerous females.

**A. Relative Fecundity**	BICOSE TAG	BICOSE Snake Pit	HERMINE TAG	BICOSE 2 TAG
BICOSE TAG				
BICOSE Snake Pit	**1.47e-10**			
HERMINE TAG	3.69e-02	5.97e-02		
BICOSE 2 TAG	**3.14e-05**	**2.16e-03**	9.14e-01	
BICOSE 2 Snake Pit	**6.51e-11**	8.89e-01	4.17e-02	**9.07e-04**

**B. Carapace length (mm)**	BICOSE TAG	BICOSE Snake Pit	HERMINE TAG	BICOSE 2 TAG
BICOSE TAG				
BICOSE Snake Pit	**6.43e-06**			
HERMINE TAG	1.07e-01	1.99e-01		
BICOSE 2 TAG	**1.08e-04**	1.88e-01	5.83e-01	
BICOSE 2 Snake Pit	**5.32e-17**	**2.76e-04**	**1.36e-04**	**1.09e-08**

**Egg volume (mm^3) Early stage**	BICOSE TAG	BICOSE Snake Pit	HERMINE TAG	BICOSE 2 TAG
BICOSE TAG				
BICOSE Snake Pit	9.27e-02			
HERMINE TAG	**4.27e-03**	1.30e-01		
BICOSE 2 TAG	**4.86e-05**	1.20e-01	8.73e-01	
BICOSE 2 Snake Pit	1.06e-01	9.94e-01	1.30e-01	8.91e-02

**Egg volume (mm^3) Mid stage**	BICOSE TAG	BICOSE Snake Pit	HERMINE TAG	BICOSE 2 TAG
BICOSE TAG				
BICOSE Snake Pit	1.85e-01			
HERMINE TAG	**7.40e-05**	**1.20e-02**		
BICOSE 2 TAG	**1.58e-05**	**2.02e-02**	3.31e-01	
BICOSE 2 Snake Pit	**1.59e-02**	3.36e-01	9.00e-02	2.23e-01

**Egg volume (mm^3) Late stage**	BICOSE TAG	BICOSE Snake Pit	HERMINE TAG	BICOSE 2 TAG
BICOSE TAG				
BICOSE Snake Pit	9.65e-01			
HERMINE TAG	**8.31e-04**	**4.08e-04**		
BICOSE 2 TAG	**2.73e-04**	**1.23e-05**	2.20e-01	
BICOSE 2 Snake Pit	**3.59e-02**	**1.99e-02**	**2.35e-02**	**1.88e-02**

Table S1. Specific p-values of Dunn tests Interannual multiple comparisons of *Rimicaris exoculata*

**A. Relative Fecundity**	*R. exoculata* TAG	*R. exoculata* Snake Pit	*R. chacei* TAG	*R. chacei* Snake Pit
R. exoculata TAG				
R. exoculata Snake Pit	**1.30e-04**			
R. chacei TAG	**7.83e-07**	**2.30e-02**		
R. chacei Snake Pit	**4.29e-05**	**3.88e-02**	9.32e-01	
R. kairei Kairei	**8.43e-08**	2.91e-01	1.39e-01	1.31e-01

**B. Carapace length (mm)**	*R. exoculata* TAG	*R. exoculata* Snake Pit	*R. chacei* TAG	*R. chacei* Snake Pit
R. exoculata TAG				
R. exoculata Snake Pit	**1.52e-10**			
R. chacei TAG	7.53e-01	**4.52e-03**		
R. chacei Snake Pit	9.11e-01	**1.26e-02**	9.48e-01	
R. kairei Kairei	**8.77e-19**	1.34e-01	**4.98e-05**	**5.60e-04**

**Egg volume (mm^3) Early stage**	*R. exoculata* TAG	*R. exoculata* Snake Pit	*R. chacei* TAG	*R. chacei* Snake Pit
R. exoculata TAG				
R. exoculata Snake Pit	0.037			
R. chacei TAG	-	-		
R. chacei Snake Pit	-	-	-	
R. kairei Kairei	0.054	0.724	-	-

**Egg volume (mm^3) Mid stage**	*R. exoculata* TAG	*R. exoculata* Snake Pit	*R. chacei* TAG	*R. chacei* Snake Pit
R. exoculata TAG				
R. exoculata Snake Pit	1.76e-01			
R. chacei TAG	**4.58e-02**	**6.57e-03**		
R. chacei Snake Pit	-	-	-	
R. kairei Kairei	**4.15e-04**	1.59e-01	**4.45e-05**	-

**Egg volume (mm^3) Late stage**	*R. exoculata* TAG	*R. exoculata* Snake Pit	*R. chacei* TAG	*R. chacei* Snake Pit
R. exoculata TAG				
R. exoculata Snake Pit	**0.039**			
R. chacei TAG	**0.004**	**0.019**		
R. chacei Snake Pit	**0.0002**	**0.002**	0.936	
R. kairei Kairei	-	-	-	-

Table S2. Specific p-values of Dunn tests multiple comparisons between *Rimicaris* species

References

Hernández-Ávila, I., Cambon-Bonavita, M.-A., Sarrazin, J., & Pradillon, F. (2021). Population structure and reproduction of the alvinocaridid shrimp Rimicaris exoculata on the Mid-Atlantic Ridge: variations between habitats and vent fields. *BioRxiv*, 3–5.

King, M. (2007). *Fisheries Biology, Assessment and Management*.

Methou, P., Hernández-Ávila, I., Cathalot, C., Cambon-Bonavita, M.-A., & Pradillon, F. (2021). Population structure and environmental niches of Rimicaris shrimps from the Mid Atlantic Ridge. *BioRxiv*. https://doi.org/10.1101/2021.06.27.450074
